# Considerations for an integrated population health databank in Africa: lessons from global best practices

**DOI:** 10.12688/wellcomeopenres.17000.1

**Published:** 2021-08-23

**Authors:** Jude O. Igumbor, Edna N. Bosire, Marta Vicente-Crespo, Ehimario U. Igumbor, Uthman A. Olalekan, Tobias F. Chirwa, Sam M. Kinyanjui, Catherine Kyobutungi, Sharon Fonn

**Affiliations:** 1 School of Public Health, University of the Witwatersrand, Johannesburg, Gauteng, 2193, South Africa; 2 African Population and Health Research Centre, Nairobi, Kenya; 3 Nigeria Centre for Disease Control, Abuja, Nigeria; 4 School of Public Health, University of the Western Cape, Cape Town, Western Cape, South Africa; 5 Warwick-Centre for Applied Health Research and Delivery (WCAHRD), Division of Health Sciences, Warwick Medical School, University of Warwick, Coventry, UK; 6 KEMRI Wellcome Trust Research Programme, Filifi, Kenya

**Keywords:** Data sharing, open science, databank, ethics, population health

## Abstract

**Background: **The rising digitisation and proliferation of data sources and repositories cannot be ignored. This trend expands opportunities to integrate and share population health data. Such platforms have many benefits, including the potential to efficiently translate information arising from such data to evidence needed to address complex global health challenges. There are pockets of quality data on the continent that may benefit from greater integration. Integration of data sources is however under-explored in Africa. The aim of this article is to identify the requirements and provide practical recommendations for developing a multi-consortia public and population health data-sharing framework for Africa.

**Methods: **We conducted a narrative review of global best practices and policies on data sharing and its optimisation. We searched eight databases for publications and undertook an iterative snowballing search of articles cited in the identified publications. The Leximancer software © enabled content analysis and selection of a sample of the most relevant articles for detailed review. Themes were developed through immersion in the extracts of selected articles using inductive thematic analysis. We also performed interviews with public and population health stakeholders in Africa to gather their experiences, perceptions, and expectations of data sharing.

**Results: **Our findings described global stakeholder experiences on research data sharing. We identified some challenges and measures to harness available resources and incentivise data sharing.  We further highlight progress made by the different groups in Africa and identified the infrastructural requirements and considerations when implementing data sharing platforms. Furthermore, the review suggests key reforms required, particularly in the areas of consenting, privacy protection, data ownership, governance, and data access.

**Conclusions: **The findings underscore the critical role of inclusion, social justice, public good, data security, accountability, legislation, reciprocity, and mutual respect in developing a responsive, ethical, durable, and integrated research data sharing ecosystem.

## Introduction

The public and population health research and development landscape in Africa has seen an increase in publications and the maturation of mostly donor-funded development programmes, research projects and multi-disciplinary capacity building networks ^1– 9^. These programmes collect and generate data that could be collated, integrated, or triangulated to address the complex and inter-related public and population health challenges in Africa. Health research data collation and sharing programmes are already in place in many high-income countries. Examples include the BigData@Heart platform of the European Union’s (EU) Innovative Medicine Initiative ^10^, the EU’s Horizon 2020 Project and Open Science Cloud ^11^, and others ^12– 15^.

The growth of databanks and repositories has expanded opportunities for data sharing to advance global health. These platforms ^16^ are setup to generate evidence-driven translation of research ^10^ which enhance our understanding of and response to public health challenges. This, in turn, can improve public health training and service delivery, and speed up health innovation. Health data integration and use is equally important in strengthening health systems. It can generate evidence-informed solutions; inform the roles and choices of patients and service providers; spur discovery to improve patient care; and help evaluate the outcome of health services and health capacity and research building programmes ^17^.

Despite the improvements of the last decades, Africa still lags behind in research and development - contributing less than 2% of global research output ^18^. While the reasons are manifold ^19^, the situation is compounded by the lack of (or limited) African-led databanks or data repositories platforms. This hampers data sharing, reuse, integration, meta-analyses, and cross-referencing. Digitisation, integration, and information sharing may allow Africa to generate knowledge more rapidly to address its public health challenges.

A vision of an African integrated databank is mindful of related challenges. These include data privacy, malicious use of data, complexities of regulating digital information, fragmented privacy regulations and jurisdictional nuances, and lack of acknowledgement of researchers and scientists ^20– 25^. Additionally, conventional informed consent and human research ethics committees (RECs) must consider emerging issues of data stewardship such as the longer storage, sharing, re-identification and indeterminate future use of collected data ^26– 30^.

The main objective of this article is to provide practical recommendations and requirements to support the development of a multi-consortia public and population health data sharing framework for Africa. This research seeks to inform a platform that will harnesses available resources, incentivise data sharing, and optimise the progress made by different research groups in Africa. The review draws on a collection of global best practices and policies. With this research, we address the challenges and misconceptions of data sharing in Africa. The collection of global stakeholder experiences on research data sharing presented here offers essential discussion points for consideration in developing an integrated population health databank in Africa. This article, therefore, targets all who are impacted by research data sharing or stand to gain from an understanding of the key tenets to consider when sharing research data in the context of privacy, confidentiality, information security and respect of human data and biological specimens.

## Methods

### Narrative review

We undertook a narrative review of publications and policy documents on data sharing in public and population health.

The methodological standards of narrative reviews described by Greenhalgh *et al.* ^31^ and noted as best suited for exploring broad and complex topics using a constructivist philosophy ^32^ were followed. Inclusion of policy documents in this review is a common practice under these circumstances ^33^. Inclusion of policy documents is also informed by the strong policy foundation of the topic, and the expectation that this review may inform future policies on data sharing. We searched eight databases for publications, namely PubMed, EMBASE, PsycINFO, Joanna Briggs, The Cochrane Library, EBM reviews, Scopus, and Web of Science. We did not set any time frames so as to include historic patterns, which may inform current data sharing practices. Our data search included all articles related to “population health data sharing” and “public health data sharing”. We also followed-up articles cited in the papers we identified in our initial search to ensure relevance of the review to our target audience ^31, 34^. The search process was, therefore, an iterative snowballing exercise.

Our initial search identified 3825 articles that were loaded into Mendeley to remove duplicates. Two independent reviewers (JOI and ENB) evaluated the title and abstract of each article to assess its relevance for inclusion in our review. This approach did not rely on a pre-defined keyword search to identify conceptually and empirically relevant documents. Any disagreements between the reviewers were resolved through discussions among the review team. We followed a qualitative appraisal based on principles of pragmatism, pluralism, historicity, contestation and reflexivity ^31, 34^. At the end, we identified 655 documents for further review.

The Leximancer software © Version 5 enabled content analysis and selection of a sample of articles for detailed review ^35, 36^. Leximancer like alternative software (such as Nvivo and MXQDA) are all paid-to-use software with limited trial period. Leximancer identifies lexical co-occurrence of natural language into semantic patterns ^37^. It is reproducible and uses an unsupervised machine learning model that is built on Bayesian Theory to predict events based on an observed pattern ^35, 37^. Leximancer identified seven core themes from the 655 articles selected. We extracted and reviewed articles with the highest co-count and likelihood of containing each theme in their segments. We selected as many as 20 articles per theme based on our reaching saturation after reading on average, the top 15 articles. Our selection of articles also involved full-text screening.

### Interviews with key informants

To ensure that our approach to the literature addressed the concerns and questions of local African stakeholders, we had interviews with 35 key informants from African-led research and capacity building programmes who produce population and public health data that could be included in a shared database. To identify these consortia we took advantage of the range of African-led programmes funded by the Alliance for Accelerating Excellence in Science in Africa (AESA) ^38^. Participants were purposively sampled, which created a diverse group, ranging from basic science and genomics to applied translation science. In-depth interviews of about 60 to 90 minutes were conducted virtually using Microsoft teams. We used an open-ended guide (see *Extended data* ^39^) to facilitate the interviews, but the discussions were flexible, with the interviewee responses shaping the discussions. We obtained a written consent to participate in the interviews from the participants. Eleven out of the 35 participants declined being recorded and notes were taken during their interview. Twenty-four interviews were audio-recorded and transcribed, and summary of emerging themes were discussed with the participants at the end of each interview. Summaries from all interviews were compiled into key themes and sub-themes. The finding of interviews presented in this paper are highly consolidated and pose no risk to the expert informants interviewed; therefore, ethical approval was not required to be obtained.

In all, the views expressed in the paper are completely based on review of literature that is available in public domain. The informal and internal consultations with network peers that constituted the interviews were used to position our findings. The consultations were also to ensure the literature review’s regional relevance, and to promote objectivity and reflexivity in our analysis and interpretation of findings. The interviews, literature review and initial analysis were conducted by two of the authors (a male and a female) with PhD in Public Health and Medical Anthropology, respectively. They have training and experience in qualitative research, ethics, epidemiology, and data science. 

## Results

The outcome of the interviews framed our approach to the meta-synthesis in the narrative review. Key observations from these discussions indicated a strong interest in research data sharing; inadequate awareness and misunderstanding of the ethical, legal, and social implications of data sharing; and pervasive data sharing between researchers based on professional and social networks. We also observed the respondents’ perceived lack of capacity for secure and responsible data sharing in the region; notable data access challenges; misconceptions of funders’ expectations of data sharing; strong fear of data misuse and exploitation; concerns about insufficient regulation and governance; and inadequate incentives and acknowledgment of data custodians.

Our analysis of the document review suggested five overarching themes: (a) Data sharing context; (b) Laws, regulations, and oversight; (c) Enablers of data sharing; (d) Governance and value-based implementation; and (e) Data infrastructure, quality, storage, and security.

Below, we present global best practice under each of the themes and discuss this in relation to the findings from our interviews with the 35 African researchers, research administrators and ethics committee members. We conclude by making recommendations to support the establishment of an integrated population health databank in Africa.

### Data sharing context

***Databanks and standards.*** Databanks or data repositories are being established globally. Notable public health database programmes feeding into repositories in the Global South include the USAID-funded Demographic and Health Surveys (DHS) ^40^, UNICEF’s Multiple Indicator Cluster Surveys (MICS) ^41^, the International Network for the Demographic Evaluation of Populations’ (INDEPTH’s) Health and Demographic Surveillance System (HDSS) ^6^ and Human Heredity and Health in Africa (H3Africa) ^42^. These platforms offer best practice standards for data sharing. The Public Population in Genomics (P3G) consortium is another global best practice model whose vision is to increase the power of analysis and discovery through greater integration. Similar and complementary protocols are available from Genome-Wide Association Studies (GWAS) Policy and the database of Genotypes and Phenotypes (dbGaP) ^43– 45^.

Lessons from genomic biobanks offer guidance on starting up future databanks ^46, 47^. These include ensuring sustainability, managing jurisdictional obstacles, governance, quality management, material transfer agreements, use of technology and intellectual property ^47, 48^. Our findings are cognisant of nuanced and substantive differences in data types and variations in the ethical and legal contexts of these data. 

Africa does not have the kind of robust, integrated databanks or data repositories present in most of the developed world. But there are opportunities to integrate existing data platforms. There is a spread of health and demographic surveillance system sites, routine national surveys, priority disease specific registries and databases, and the proliferation of genomic data repositories in the region ^6– 9, 42^. Other examples include routine DHS, large scale donor funded research and/or development programmes across the continent, country specific survey and administrative datasets, and data emerging from the Developing Excellence in Leadership, Training and Science in Africa (DELTAs Africa) programme.

INDEPTH – one of the oldest data platforms in Africa offers good data sharing practices. It provides potential to collate data from member HDSS sites into outputs that enable systematic comparisons ^6^. Another example is the H3Africa programme which provides exemplary lessons for an integrated African databank ^42^. The H3Africa consortium conducts biannual research priority setting and regular review of operational policies, guidelines, and logistics. These measures are essential for standardisation and quality assurance ^42^. In all, Africa has pockets of quality data that may benefit from greater integration.

***Perceived challenges, risks and considerations for data sharing***. Individual willingness to share data is mediated by sociodemographic status, cultural and religious factors ^49– 54^. For example, younger people and females are less likely to participate in consenting to data reuse ^55^. Fears of loss of privacy or confidentiality breach, commercialisation of data, misuse and abuse are equally concerning ^56– 59^. These concerns are also driven by insufficient public engagement and low public awareness of research governance, participant protection and risk minimisation measures ^54^. This leads to minimal public appreciation of the importance of health research. 

Poor communication and use of technical terms may breed mistrust and impede participation and willingness to permit data sharing ^60^. The use of language and analogies that are sensitive to the context of research could improve communication and understanding ^61^. In addition, studies have raised concerns about participants’ understanding, and the quality and extent of information participants should have in order to make informed decisions ^62, 63^. To deal with this problem, authors recommended improving study participants’ knowledge of data sharing ^61, 63^ with tools such as videos ^64^, pictures ^65^ and vignettes ^66– 69^.

Beyond research participants, our findings highlight that scientists are concerned that the risks of data sharing might outweigh the advantages. This perception is driven by the fear of possible loss of academic advantage and independence; the possibility of their work being misused, misinterpreted or misrepresented; the loss of intellectual property; and an increased workload for administration and data management ^70^. If these issues remain unaddressed, the practice of data sharing will remain a dream in Africa. Major funders of public and population health research in Africa expect that data sharing should be the norm ^71– 77^. In most cases, funders provide global tools for sharing data ^78, 79^. We, however, found no evidence of donor support in terms of financial resources, capacity building or infrastructure to facilitate an African integrated interdisciplinary data custodial and sharing mechanism.

Other important risks of data sharing include concerns of data quality; poor curation and indexing of datasets; variations in data provenance, metadata and management protocol with implications for data comparison and integration of datasets and databases ^80^. Most of these challenges may be addressed through rich collection of metadata of each data set ^80, 81^.

Relatedly, trust in databanks ^82^ is dependent on the perceived trustworthiness of the data custodian ^83– 85^, use of minimum set of information provided ^84, 86– 89^, and the promise of, and belief that privacy will be maintained ^84– 87, 89^. Without these elements there is no public trust.

Factors affecting public attitudes to data sharing have been summarised as sensitivities, controllability, benefits, risks, governance and public attitude ^53^.

Internal policies, collaborative agreements and contracts within research networks and specialised fields of public and population health govern data access and sharing are essential elements of data governance ^90^. These instruments are, in part, designed to mitigate some of the challenges. 

### Laws, regulations, and oversight

***Data protection laws.*** As of 2018, only 19 African countries had privacy protection laws ^91^. Six others (Kenya, Nigeria, Togo, Tanzania, Uganda and Zimbabwe) had laws in draft stages. An analysis of the privacy protection laws across the continent classified almost all of these laws as moderate to limited ^92^. Whatever differences may exist between countries, within-country variations in privacy regulations is equally common ^93^. Consequently, countries have developed mechanisms to facilitate lawful application of their, often conflicting and fragmented, privacy regulations ^24^.

For African countries without privacy protection regulations, there are global models to explore. These include the UK Data Protection Act of 2018 ^94^ (see principles in Box 1) and examples from the African continent ^92^. These tools give individuals control of their data through their right to informed consent ^56^. They also stipulate special protection for certain types of data including genetic and biometric data ^95^.

**Box 1. UK data sharing principles**

1. Personal data shall be processed fairly and lawfully and shall not be processed unless – (a) at least one of the conditions in Schedule 2 is met, and (b) in the case of sensitive personal data, at least one of the conditions in Schedule 3 is also met.

2. Personal data shall be obtained only for one or more specified and lawful purposes and shall not be further processed in any manner incompatible with that purpose or those purposes.

3. Personal data shall be adequate, relevant, and not excessive in relation to the purpose or purposes for which they are processed.

4. Personal data shall be accurate and, where necessary, kept up to date.

5. Personal data processed for any purpose or purposes shall not be kept for longer than is necessary for that purpose or those purposes.

6. Personal data shall be processed in accordance with the rights of data subjects under this Act.

7. Appropriate technical and organisational measures shall be taken against unauthorised or unlawful processing of personal data and against accidental loss or destruction of, or damage to, personal data.

8. Personal data shall not be transferred to a country or territory outside the European Economic Area unless that country or territory ensures an adequate level of protection for the rights and freedoms of data subjects in relation to the processing of personal data

*Source: Government of UK Legislation. Data Protection Act 2018. http://www.legislation.gov.uk/ukpga/2018/12/contents/enacted.*

***Ethics committees.*** Ethics committees include research ethics committee (REC), biomedical research ethics committees (BREC) or institutional review board (IRB). In this article, we use the term research ethics committee (REC). These are multidisciplinary, independent groups of individuals appointed to review proposed studies with human participants. The REC ^96^ must ensure respect for participants; beneficence, as well as justice by protecting their rights, safety, and well-being.

The composition, structure and requirements of RECs vary between countries. Some countries require additional permission or registration to conduct research. However, RECs have a role to play in the transfer of data to a third-party institution by ensuring compliance with data control regulations and privacy protection policies.

Yet, in many countries, RECs are confronted with numerous challenges including lack of legal protection ^97^, inability to reach quorum in decision making, inappropriate constitution of REC ^97, 98^ and inefficiency or bias amongst its members ^99^. In addition, the growing scope of social implications of data sharing often falls outside the responsibility of RECs whose adjudication is based on presented intention of a particular research project without detailed consideration of broader social impact of the research ^50, 100, 101^.

Fortunately, there are a number of global guidelines to rely on for direction even if most RECs have not kept up with recent developments in research and technology. The Helsinki Declaration remains a major reference document for data security, ethical principles and governance of data sharing ^102^. Others include the Australian Guidelines on Human Biobanks and Genetic Research Databases ^103^; The OECD Principles and Guidelines for Access to Research Data from Public Funding ^104^; the Bermuda Principles ^105^; and the Expert Advisory Group on Access (EAGDA) report on Data Access ^106, 107^. Similar tools have been developed in parts of Africa ^108^.

***Consent.*** Informed consent is the cornerstone of ethical conduct and regulation of research. Increased digitisation of health data has resulted in easier access to data, and data integration facilitated by greater connectivity via the internet ^80^. This calls for more attention to the ethical and legal implications ^109^. The universally applicable guidelines for consenting involves three key features: (a) of information to potential research participants needed to make an informed decision; (b) facilitating the understanding of what has been disclosed; and (c) promoting the voluntariness of the decision to participate or not in the research and ensuring respect for participants. Ensuring that the informed consent process fulfils these three requirements can go a long way towards mitigating problems.

For data to be shared for further future use, RECs need to issue waivers permitting the use of de-identified data or broad consent from research participants ^110^, as well as contending with emerging considerations of data stewardship such as the longer than usual data storage, sharing, re-identification and indeterminate future use of collected data ^26– 30^. These approaches have their limitations. For instance, the proliferation of data sources and hubs increases the risk of unlawful re-identification. Different consent options are described in detail in terms of their benefits and risks by Peppercorn *et al.* ^111^.

Dynamic consenting allows research participants to opt-out or opt-in at different stages of the research after the original informed consent was issued ^112– 115^. On the other hand, broad consent impede participants’ control of their data ^116^. From the participants’ perspective, realistic measures to allow dynamic consenting should be detailed in the original consent. Re-contacting participants should of course, follow standard ethical principles including options on communication of findings or participant access to data ^117, 118^.

Further, it has been suggested that the respect accorded to study participants or groups during primary data collection should be maintained in secondary data storage, sharing and reuse. Elements of respect include privacy protection and confidentiality; autonomy; data security; respect for individuals and group rights; ensuring dignity of participants; and, protection of life, wellbeing and welfare ^10, 102, 112, 119^. In this regard, any further use of data should be in line with the scope of original informed consent provided by the research participants. To mitigate likelihood of unknown future use, authors have pointed out that participants must be subjected to appropriate informed consent as discussed above. In the case of specific consent, the intention of the research is clearly stated at the time of data collection including likely future use of the data ^112, 114^. In the absence of this certainty at the time of data collection, broad consent may be adopted with conditions to protect the research participants ^112– 114^. Such protection may be offered by RECs or data access committees. It is still incumbent on researchers to provide as much information as possible when broad informed consent is solicited.

Reaching a consensus on data sharing practices and data reuse has not been systematically addressed, particularly in Africa. Other important yet unaddressed issues include public views or perceptions of cross border data transfer ^120^. The differences in jurisdictional powers of national governments and other oversight institutions such as RECs seem to be part of the impediments. Other considerations for the deployment of a data sharing platform include identifying data sources/patterns, engagement with leaderships, ethical and regulatory compliance, data management and legal conditions ^121^.

Ethics waivers have been given for data reuse in circumstances where it is impossible to obtain informed consent ^102, 112, 114, 117^. The RECs determine the reasonability of circumstance for waiver ^117, 122^. Such waivers should preclude secondary use of data where participants are identifiable ^123^. A common example may include the request for ethics waiver to use medical records of readily accessible and regular users of health services such as patients on chronic treatment. Others have cautioned against the negative psychosocial implications of re-contacting people to consent including deceased family members or reliving a past trauma or unintended breach of privacy ^120^. Additionally, researchers have argued that data collected with public funds during routine service provision should be maximised for public benefit and so support such waivers ^124– 127^. Generally, many have favoured use of aggregated data when individual consent cannot be obtained. In this context, the impact on groups or communities should be considered and similar group anonymity should be ensured if necessary ^128^. On the other hand more stringent measures to obtain ethics waivers have also been recommended ^55, 129– 131^.

***Data ownership and custodianship.*** Data ownership is very contentious especially when it comes to sharing the data. The data may be held by an individual scientist or collaborative teams; manually or digitally collected or generated; and stored locally or in shared repositories ^132^. Other aspects may be related to individuals involved in data collection, and those who store and share data. Interview with DELTAS Africa consortium stakeholders revealed a wide range perceptions on the issue of data ownership. Many consortium stakeholders argued that the funding bodies were the owners of data and had the responsibility of deciding when and how data should be shared. Others argued that the principal investigators, researchers, governments, or academic and research institutions were primary owners of these data. Few participants, including members of RECs perceived data ownership to encompass study participants and communities where studies are conducted. Given the complexity of data ownership, and that many stakeholders can mount logical argument as to ownership, scientists have recommended non-exclusive ownership of data. They submit that data ownership should be governed by legal and moral obligations including trust and custodianship with variations in the right of access and utility by different stakeholders ^133– 135^. They have argued that data ownership should be based on national privacy regulations and permission granted.

***Intellectual property rights.*** Closely linked to the issue of data ownership is intellectual property rights. Many researchers we had interviews with voted in support of a system that recognises researchers’ or scientists’ contributions and their further involvement in the use of their data if possible. Ultimately, it has been argued that this procedure should be guided by local intellectual property laws ^104, 114, 136^. Similarly, databank users are required to report back to the custodians of the databanks all publications and patents emanating from the data provided to them ^107, 117, 119^.

Authors of the reviewed documents have suggested that data sharing and implementation of databanks should be based on the principle of distributive justice by optimising benefits to society, minimising harm and equitable beneficence related to accessing data and emergent health innovations ^10, 47^. This proposition invokes the principles of transparency and equity by ensuring that benefits are shared as broadly as possible, especially when dealing with vulnerable populations ^114, 117^. Benefit sharing is extended to include equitable and fair access to the databank. Most databanks policies are, however, not limited to non-commercial use given that some commercial uses are aimed at creating public good and the distinction will determine access.

### Enablers of data sharing

***Trust and transparency***. Gaining and ensuring the trust of individual research participants and the public has been described as an essential element in building and maintaining databanks ^10^. Trust is a by-product of different principles of good research ethics including clear consultations, open communication and recognition of the individual’s autonomy ^137, 138^. In the case of big databanks, authors have suggested that these attributes should be on-going and not a one-time checkbox activity. Maintaining public trust facilitates benefit optimisation, promotes respect, mitigates harm, and enables social justice and priority setting. Trust may be derived from involving the participants and civil society representatives in the design, governance, knowledge translation and beneficiation of the databank output ^139^. The engagements should also be cross cutting to involve other researchers, policy makers and funders ^112, 113, 140, 141^.

Transparency helps to build trust and accountability and may be achieved by allowing inclusive stakeholders access to policy, guidelines, and data sharing operations. Research participants expect a transparent platform to be clear about how data will be shared and with whom ^53, 142^, the type of research that is to be performed ^143^, by whom the research will be performed, information on data sharing and monitoring policies and database governance, conditions framing access to data and data access agreements ^144– 146^, and any partnerships with the pharmaceutical industry ^147^. Patients and research partners are also interested in knowing how involved patients and other human rights advocacy groups will be in providing oversight and supervision of the platform to ensure unbiased access and use of the databank ^148^. Transparency may be enhanced by keeping and communicating sufficient records of operational activities including audits logs and trails ^86, 87, 149, 150^; notification of study participants when records are accessed ^84, 86, 151^; operating a decentralised data storage system ^87^; and use of data for only specified and agreed purpose ^86– 88, 152^.

***Stakeholder and community engagement***. The success of data storage and sharing is dependent on inclusive stakeholder engagement ^10^. Engagement facilitates fair negotiation and consensus on thorny issues. Authors recommend that community engagement should start at the beginning of the project. While our list is not exhaustive and may vary with the type of research conducted, some of the key stakeholders to consult or engage with may include the study participants or patients, civic organisations and leaders, government departments heads of relevant parastatals and nongovernmental organisations, academic research administrators, ethicists, established researchers, graduate students, industry representatives, human rights lawyers, clergy, and traditional leaders.

Stakeholder consultation is an important strategy to promote other essential elements of data storage and sharing such as equity, trust, transparency, autonomy and participation ^10, 109, 153^. For example, H3Africa provides a framework for community engagement ^154^. The key components in this framework include defining the goals of engagement; defining “the community” or “the public” in research; identifying strategies, models, and methods for community engagement (e.g., consulting gatekeepers, community meetings); identifying who will do the engagement as well as outlining the role and expectations of community engagement.

The Tikanga Framework of New Zealand, aimed at including Maori People in decisions regarding the use of their data, is an example of a flexible system that is responsive to the material circumstances of its target population ^96^. Databanks may need to tailor-make their standard operating procedures to address the unique needs of specific groups ^155^. It is important to ensure continuous and appropriate interaction with stakeholders.

Engaging marginalised and vulnerable populations is one of the cornerstones of developing an effective databank. Therefore, measures to promote greater participation of these groups are recommended ^156^. In addition to the importance of trust, it is suggested that improving the relationship with the public enhances their disposition to information and sample sharing, minimises common concerns and increases public participation ^157^. Consequently, authors have recommended that from the onset of projects, researchers should have a clear plan to involve their target community in the development of the implementation and accountability measures including opportunities to learn about the databank, measures to regularly update the public and ways of addressing concerns about the databank ^157^.

***Incentivisation of data contributors and users***. In reality, scientist are not as forthcoming with their data as expected ^158– 163^. Similarly, there are divergent views on the extent of data sharing among researchers and reported variations are contingent on career ranking and years of experience ^159, 164^. This difference may be associated with professional disciplines. In life sciences, geneticist are more likely to deny others data when compared to non-geneticists ^160^. This is due to variances in intra-disciplinary data collection protocols, sharing requirements and expectations. Nationality of researchers was also a factor likely to effect the prevailing local data sharing culture ^159^. Some of the reasons why scientists withhold data include funding agreements, collaborative agreements, data sensitivity, privacy, giving up chance to publish, public critique, lack of data repositories and the absence of consent to share ^160, 165^. The scepticism about the benefits of data sharing is also common among researchers. Furthermore, researchers in low resources countries fear that their data will be exploited by better resourced scientists ^161^. Others view data sharing as a threat to intellectual property, professional value and economic benefits ^166^. The greater value placed on publications by institutions has the potential to discourage data sharing ^164^.

Best practice solutions suggested by authors include human capital and infrastructural development, and financing to promote research data sharing ^165, 167– 169^. Tangible reward in the form of reputational incentives and peer recognition including citation may promote data sharing ^158, 170^. Increasing visibility of open access data may also promote sharing ^158^. Additionally, creating incentives in the form of rewards may promote data sharing by scientists ^46, 158, 171^. One example is the Cochrane-REWARD prize for reducing waste in research ^172^.

Data sharing may be more effective if it is a requirement of the funding agreement. This is particularly important as African scientists view funding agreements as an obstacle to data sharing. Nevertheless, this view is contrary to the expectations of most funders of research in Africa ^72– 77^. A public list of funded entities and the data they hold could be made available to promote data sharing and reuse. Policy enforcement may not be sufficient to ensure data sharing and there is need to for a cross-institutional community of practice to promote collaboration and sharing ^71^.

Network and co-citation analysis may be used to promote the visibility of available datasets to scientists working in similar fields. Such efforts should be supported with a clear policy that addresses the concerns of all stakeholders, including monitoring and reward mechanisms ^161, 173^.

Nomenclature, metrics, and weighting of data source citation like citation of peer reviewed publications should be considered. This proposition resonates with the San Francisco Declaration on Research Assessment ^174^. Further recommendations of how this may be realised are described by Jones *et al*. ^71, 175^, including the recommendations of DataCite Collaboration ^176^. Additional guidance is provided by the Joint Declaration of Data Citation Principles (JDDCP) ^177^.

Promoting international collaborations and publications may be seen as added incentives, as it may unlock global recognition and additional funding opportunities ^178^. Lastly, open data badges are the only known tested intervention to improve data sharing ^171, 179^. Expressly, evidence on effective rewards for data sharing remains unknown and under explored.

***Funders’ and researchers’ position***. Findings from our interviews with African stakeholders showed that most researchers or scientists in Africa were hesitant to share their data largely due to lack of awareness of the benefits of data sharing, similar to findings from reviewed documents. We also found that many researchers, especially in low-and-middle income countries (LMICs) fear of loss of academic advantage/independence; and the possibility that their work may be misused, misinterpreted or misrepresented among many other reasons ^161, 166^. Some consortium researchers also believed that research funders restricted them from sharing data. Contrary to such beliefs, the Wellcome Trust presents a summary of funders’ statements on data sharing as it “expects all of its funded researchers to maximise the availability of research data with as few restrictions as possible” ^180^. The summary excluded the more recent USAID’s Policy on Development Data ^181^, which purports that “data, and the information derived from data, are assets for USAID, its partners, the academic and scientific communities, and the public at large. The value of data used in strategic planning, design, implementation, monitoring, and evaluation of USAID’s programs is enhanced when those data are made available throughout the Agency and to all other interested stakeholders, in accordance with proper protection and redaction allowable by law”. As such, we recommend proactive advocacy to ensure that the concept of data sharing becomes a mainstream consideration in national discussions of research management and governance ^70^.

The above issues may be amenable to the roles and functions of RECs as an unbiased and value-based entity to arbitrate lawful and moral use of data. However, there were questions about whether most members of African ethics review boards are familiar with the concept of data sharing amongst other ethical issues discussed such as broad consenting. This is similar to what we found in our interviews with DELTAS Africa members including REC members. REC participants recommended that their members be trained and provided with opportunities to attend workshops or other platforms that can expose them to new trends on data and data sharing.

### Governance and value-based implementation

***Policies and values.*** Most guidelines and regulations in Africa do not provide clear guidance on governance and how data and biological specimens ought to be shared ^182, 183^. This is particularly critical given that the different actors involved in data sharing may have different perspectives on data. For example, research participants may be concerned about confidentiality, how the data will be used, and how they might benefit. On the other hand, data collectors may want to produce high-quality data, while data users aim to advance science and inform policies. Clear examples can be borrowed from the UK, USA and Canada. All regulations offer opt-out options when using data for research other than the original intention it was collected for, with the UK National Data Guardian’s recommendation being more stringent ^24, 184^. The European Union General Data Protection Regulation of 2016 ^185^ has also been hailed as an effective framework to facilitate regional harmonisation ^24^. Sector-specific guidelines have been recommended to promote pragmatic compliance with policy.

Given such differences, there is need for data sharing policies to state clearly when, where, how and which data should be archived and made available.

Lack of clear policies on data sharing may frustrate researchers who want to share data, and provide loopholes for those who are unwilling to share. Thus, in the absence of absolute privacy protection, risk minimisation is the best alternative ^58, 186^.

Awareness of risks did not always affect willingness to share data when such risks were weighed against expected benefits ^53^. Hence, willingness to share data was more likely to become a factor of “privacy – utility trade-off” ^187^. Similarly, most privacy protection regulations do not consider privacy as an absolute right of an individual but contingent on its intersection and weighting against other rights ^24^, for instance, the imperative to report a notifiable disease or in case of the safety of children and vulnerable people ^188^.

Greater integration also poses risk of re-identification, which infringes on participants or patient privacy protection and trust. This is a major concern for people who share data ^57, 58, 133^. Likewise, the willingness to share data decreased with increase in privacy and confidentiality concerns ^52^. Criminal prosecution for negligence or wilful breach of privacy as stipulated by national laws should be considered. Various recommendations for privacy protection have been made including creation of clear laws to govern re-identification, and stronger sanctions and corresponding enforcement protocol for misuse of data ^133, 189, 190^. The use of data without following due process or attribution should be condemned ^46^. In all, the risk of re-identification continues to rise and might as well be recognised, regulated, and used to serve public health interest.

***Data anonymisation and re-identification.*** The protection and access to data should be reasonable to allow maximisation of the databank. As a consequence, there are limitations to anonymising data ^112, 117^. Anonymity will not allow linking datasets and growth of the database may depend on re-identify individuals if there is ethical reasonability and lawful approval to re-identify the participants ^113, 119^. Regardless, the principle of privacy protection must be always upheld, and such measures should be sufficiently described in the protocol for ethics approval. The data reuse options, and protective measures should also be detailed in the informed consent to involve participants in the decision regarding the reuse of their data by the researcher or a third party. These permutations make a fallacy of absolute anonymity. Hence, the growing call to inform participants that absolute anonymity is increasingly impossible to guarantee ^107, 191, 192^. The difficulties of absolute anonymity are well described ^193^. It has, for instance, been demonstrated that surnames can be re-identified using gene sequencing data ^194^. Special training or augmentation of existing human research ethics curricula on the use of secondary data may be warranted, and certification mandatory in the event of inter-researcher data sharing.

Understanding the differences in maintaining anonymity is essential to guard against infringement of privacy. Thus, distinctions are made between anonymisation ^1^, identifiability ^2^ and re-identifiability ^3^ ^137, 195^. There is also the concept of pseudo-anonymisation; this involves removing identifiers and replacing them with single or double blinded codes to anonymise the data in a way that will allow authorised re-identification if or when there is ethical or legal imperative ^95, 196^. 

The reality is that patients’ data are shared across departments for clinical care and for billing purposes. There is also an increase in clinical audit of patient records for quality improvement of practice and research without individual patient consent or promise of anonymity by researchers ^50, 198– 201^. Similarly, social media is increasingly being used to mine vast biopsychosocial and other personal data, sometimes without authorization or consent of the individuals whose data is being used ^202– 205^.

Recognition of these realities, complemented by better regulation should mitigate unintended consequences such as stigmatisation of individuals or communities, genetic discrimination, racial stereotyping and discrimination, commercial exploitation of vulnerable groups, legal jeopardy and shaming ^120, 206, 207^.

Various measures to ensure anonymisation of data have been proposed ^208^. An essential step is to become aware of possible identifiers, which can be direct or indirect ^209^. Malin *et al.* provide re-identification risks assessment and mitigation measures ^191^. 

Some ethical issues to note in relation to re-identification or computational phenotyping of data without participant consent is that it may constitute an infringement to the principles of autonomy and respect for person, beneficence and justice ^210^. This makes re-identification a double-edged sword requiring due consideration. Re-identification without authorisation takes away a person’s right to decide – this may extend to inferences or attributions being made about a dataset based on attributes from an unmasked data set. Equally significant is the re-identification and use of data of minors with consent and assent ^210, 211^. Re-identification or computational phenotyping may create an undue attention to a group or individual in a manner that may incite or perpetuate unfair treatment ^212– 215^. A lot of these challenges may be addressed by upholding the consent given by patients or study participants, use of appropriate technologies, mechanisms and permission to promote pragmatic dynamic consenting processes ^216^. Over regulation of the data should also not become an impediment to robust scientific work ^217^.

Some studies have recommended the sharing of random subsets of the database stripped of all possible individual unique identifiers ^153^ or to use aggregate datasets ^218^. Other authors have suggested the inclusion of noise elements in aggregate data to further mask the dataset ^191^. The noise elements may be in the form of random value changes, data swapping (switching values in the record), and synthetic data generation (creation of data from attributes of real records without corresponding to any real individual).

***Data access control***. Access to collected data may be open, controlled or hybrid depending on the level of sensitivity of the data and privacy concerns ^166, 193^. Open data is available for anyone to use without permission. However, controlled access data requires special permission. Controlled data have higher risk of individual data re-identification and access to it may be made by the data access committee once all safety measures are met. The hybrid model combines both methods with restricted and open access to some data, thus, it carries a lower risk of re-identification of individual participant data. Similarly access control may be centralised in a pooled data system while access may be localised to the custodian in the federated system ^166, 193^. The different approaches should not negate the principles of autonomy, privacy, public interest and benefit, acknowledgment of data contributors, transparency, accountability and trustworthiness ^193^. 

Limited awareness and access to databanks available for secondary users may decrease the return on research investment in Africa. Timely access to data is an essential requirement of data sharing governance ^219^. Access to and uptake of data should be promoted during stakeholder engagements and collaborative partnerships. This extends to devoting resources to addressing the impediments to data sharing ^220^. A review of global recommendations ^219^ indicates that access to secondary data should be determined by the nature of the material available; the purpose of the request; the need for additional ethics clearance; intellectual property agreements; user fees; ownership of material; conditions of informed consent; assurance of confidentiality; and, material or user restrictions.

As a guide to data access, Desai *et al.* ^221^ propose the following five ‘safes’: “safe project (is the use of the data appropriate?); safe people (can researchers be trusted to use it in an appropriate manner?); safe data (is there a disclosure risk in the data itself?); safe setting (does the access facility limit authorised used?); safe output (are the statistical results re-identifiable?)”. While the ‘safes’ provide a quick frame of reference for review, they should of course be used on the backdrop of local regulations, definitions and contexts. Other guides include “10 rules for responsible big data use” ^222^, and the seven recommendations of the Caldicott Commission ^188, 223, 224^. 

The decision on access to data is also based on its ethical merit, public good, level of risk and mitigation measures proposed ^153^. Other elements of the data access agreement may include “specific research objectives; plans for publication; permissions for and monitoring of access to the data; data storage, security, and confidentiality; allowances for copying or remote use, if any; de-identification plans; data destruction protocols; and, identification of parties responsible for data analysis and data security” ^153^. Others have included up to 12 months after data release to publish findings of the research ^43^.

The agreement should also prohibit users from re-identifying de-identified data without appropriate approval by an ethics committee ^43^. Intention to obtain data from other sources that may result in wilful or accidental re-identification should be carefully considered and declared. This act is described as data linkage and has been described in terms of its process, risks and benefits ^225^. There is a growing list of studies that applied various data linkage methodologies to address complex issues ^226– 230^. There are proposals on how to use anonymised linkage technologies or split file methodologies to protect sensitive information or to de-identify multiple datasets after linkage by a bona fide third party with no conflict of interest ^231– 233^.

Most data sharing agreements are silent on the consequences of violating data access agreement ^234^ and rely on national regulations. This too must be explicitly stated in the agreement. Authors suggested that non-compliant users of the databank resources (principal investigators [PIs] and their Co-PIs) should be prohibited from using the databank and reported to authorities in their institutions, funders and other regulatory authorities and databanks ^98, 235^.

### Data access committees

Access to databanks is controlled by data access committees (DAC). DACs are tasked with the responsibility of reviewing data access requests and serve as oversight committees to approve or disapprove data access applications. The committee may be made up of civic organisation representatives, PIs, funders, other researchers, representatives of the group from whom the data was obtained, journal editors, and ethicists. Their specific roles include acquiring and storing data, ensuring data protection and information privacy, ensuring compliance to research consent agreements, protecting data quality and data donors, and balancing of timely publication with open access to data ^134, 236– 238^. They equally have a fiduciary role to develop inclusive and unambiguous policies needed to execute these responsibilities.

There are two levels of governance of databanks – internal daily operations and external policy administration and stakeholder relations ^70^. Governance provides a set of standard operating procedures, and ethical and legal consideration to inform the strategic and operation management of biobanks ^239^. These principles also cover issues of funding, internal and external auditing and quality control, standard operation procedures for managing samples or data and ethical and legal consensus on management of samples and data. It is also part of the governance functions to have clear presentation processes of data collation, storage, use, and disclosure including policies and processes of data protection and risks assessment that may need to be updated regularly ^83^. Specifically, the governance function of ensuring data protection entails measures to guard against privacy breaches such an unauthorised access to data or security breaches resulting from a deliberate attack on the system leading to loss of control of the dataset in their custody. In addition, governance entails providing a guideline on who, how, when and under what authority datasets can be linked or merged ^83^.

Despite the important mandate that DACs play, they are confronted with various challenges, chief among them financial constraints and lack of sufficient oversight mechanisms ^240^. In addition, there is lack of clear definition of the relationship between DACs and biomedical RECs. In response, data custodians have pooled resources to develop a single better resourced DAC. The GA4GH provides a good framework to model from or adapt as necessary ^241^. 

Moreover, to address inequalities and curtail vested interests, authors have recommended that DACs should be inclusive, global and transparent ^242^. This approach may address the issues of trust, transparency, equity, legitimacy, integrity and accountability ^173^. In other words, DACs should be constituted to have a full spectrum of its stakeholders. To ensure fairness and effective executions of other fiduciary responsibilities, data access committee should be an independent committee without conflicts of interest and should have mechanisms to evaluate and mitigate its internal risks ^240^.

### Data infrastructure, quality, storage and security

***Data quality.*** The quality of shared data is important to ensure reproducibility ^241, 243– 247^. Scepticism and self-doubt of quality of research may inhibit some researchers from sharing their data ^178^. Data quality is a challenge in Africa due to lack of infrastructure, inadequate skills, and capacity amongst researchers as well as lack of guidelines on how data must be prepared or processed as discussed above. These concerns parallel what we found during our key informant interview with African research stakeholders.

Databanks are required to work with data contributors to establish and continuously implement data quality assurance measures including developing quality threshold indicators for routine review and updating ^104, 112, 117, 248^. Studies have reported that data quality assurance should be documented, unbiased, open to review, factual and proportionate ^10, 104, 117, 119^. African research may need to focus on generating more high-quality data. The H3Africa routine participatory process ^42^ may be a model to emulate as it assures control, compliance, and accountability along its data management value chain. While enforcement of data quality may not be enough to facilitate reuse ^249^, data seal of approval is additionally offered by repositories guaranteeing researchers that data will be stored in a measure that assures their quality and consistent reuse while ensuring the trustworthiness of digital archives ^250, 251^.

Regulatory licencing and oversight of databanks could also help ensure quality ^252^.

***Data storage and retrieval***. Integration of different datasets during storage may have risks, including re-identification of anonymised data, risk of disclosing other data, misinterpretation of data for various reasons, malicious use of data, harm to the public posed by illegal disclosure and commercialisation ^128, 253^. Cataloguing data in a consistent manner will promote harmonisation and interoperability ^254^. This is further enhanced by using internationally accepted norms and standards to ensure compatibility ^104^. Castillion *et al.* ^255^ provide a comprehensive list of the requirements for online repository to address some of the common issues on security and utility. The sub items include metadata availability, discoverability, data standardisation, quality assurance, storage, backup, migration, succession plan, legal status, access and terms of use ^161, 255^.

Most consortia have relied on data integration systems such as the Open Archival Information System (OAIS) ^256, 257^, which enables the management of organisations and individuals intending to share data. The system offers a guide for developing common terminologies and concepts, architectures and operations of databanks to facilitate uniform and valid content sharing ^258^. Detailed description of the complete enterprise system with data security features are described by Winter *et al.* ^258^.

To ensure privacy protection, most databanks store anonymised or de-identified data with additional safety and access control measures to secure the data in their custody ^24, 113, 118, 259, 260^. Strategies on maintaining anonymity have been developed above. To maintain anonymity, some studies have recommended the sharing of random subsets of the database stripped of all possible individual unique identifiers ^153^ or to use aggregate datasets ^218^. Other authors have suggested the inclusion of noise elements in aggregate data to further mask the dataset ^191^. The noise elements may be in the form of random value changes, data swapping (switching values in the record), and synthetic data generation (creation of data from attributes of real records without corresponding to any real individual) ^191^. To ensure data truthfulness in public health, two general methods of re-identification prevention are used. These are data generalisation and suppression ^191^. Under generalisation methods, data is replaced with general values and under the suppression method, unique identifiers are excluded from the data release ^261– 264^. Details for data de-identification and anonymisation measures for different data and sample types are described in a literature ^189, 194, 265, 266^. Other authors have recommended limiting time of access to datasets as well as the data they can access for a clearly defined project ^128^. In addition to the mitigation measures, some countries prohibit unauthorised re-identification of shared data ^267^.

The diverse datasets and data sources, and the technological advances in data management increase the risk of re-identification. Therefore, case-by-case consideration should be given to different requests by the data access committee and research ethics committee. Pharmaceutical industries for instance, have professional bodies and working groups (such as TransCelebrate ^268^ and Pharmaceutical Software Users Exchange ^269^) that develop and regulate policies and procedures for data de-identification. Tucker *et al.* ^260^ have summarised best practice approaches to ensure data protection recommended by relevant institutions. In addition, Jones and Ford ^253^ have proposed models of integrating administrative data with other clinical data and reported practical applications of the different models together with ethical, legal and social requirements for each model. They distinguish between two models ─ pooled data and federated data ─ by where the data is hosted and accessed. With a pooled system, data is accessed through a hosting entity whereas in a federated data model, data may be accessed through the source organisations.

The need for standardisation of data management frameworks that clarify data storage and sharing methodologies is central to both pooled and federated data sharing models. The framework may include standardisation of variable names, codes and storage format ^270^. An alternative will be to adopt a standard metadata structure to allow transformation and integration as required by a central data management team constituted by a core team and representative data managers from across the consortia ^238^. The core team may be made up of a neutral convening organisation with a governance function including convening stakeholders, quality assurance and oversight, financial management, communication, policy development and execution ^238, 270, 271^.

***Security***. The safety of the data in most countries is protected by national privacy protection regulations, such as those mentioned above, and must meet human research ethical committee standards and approval ^272^. These laws mandate the custodians of data to protect it from abuse, unauthorised access and tampering, loss or unlawful disclosure ^272^. Privacy protection stipulates a notification obligation in the event of breach of privacy due to unauthorised access, loss or disclosure of information in the care of a legal data custodian ^273^.

The three biggest cloud data storage service providers include Amazon, Google and Microsoft ^274^. This cloud computing and few service providers come with significant risks ranging from integrity and exploitation of data by the service provider and its employees ^222, 274– 276,^, cloud attacks ^277^, user identity spoofing ^278^, data tampering ^279^, denial of service ^280^, unlawful access to database and infiltration of the system ^278^, as well as re-identification of de-identified data ^281^. Lessons from adverse experiences may offer hope to mitigate some of the risks in future ^274, 282^.

Some proponents of data security favour the establishment of remote access controlled data centres with state of the art monitoring systems to avoid physical transfer of data or unauthorised access or utilisation of datasets with capabilities to provide feedback or alerts on infringements ^107, 283^. Others have recommended the use of secure encrypted servers for data transfer ^153^. They added that such electronic data transfer options should have multifactor authentication steps to access the databank with restriction to downloading or copying the dataset. Methodologies to ascertain the likelihood of re-identification are also evolving with their strengths and limitations ^234^. Examples of the methodologies include K-anonymity ^261^ and unicity ^284^. 

There are various techniques for ensuring secure sharing of electronic information ^285^. These techniques are grouped into two broad categories including the cryptographic and non-cryptographic techniques ^286– 288^. Cryptographic techniques encrypt stored data over the network and uses authentication techniques requiring decryption keys and verification using digital signatures ^285^. These systems are also capable of providing patient control over their data by granting patient encryption and decryption control to allow access users of their choice.

Protection of electronic data is an ongoing process and various mechanisms have been adopted. These include the use of patient encryption ^289^, employment of a third party to protect data integrity through layered encryption ^290^, data partitioning techniques ^291^, digital signatures ^292^, hierarchical encryption ^293^, the Elliptic Curve Digital Signature Algorithm (ECDSA), a cryptographic algorithm (used by Bitcoin), and many other techniques with their own strengths and limitations ^285^. Variant three of the ECDSA is acclaimed to withstand many of the risks already described. The choice of privacy protection techniques adopted should also be made based on its functionality and implication for data accuracy using a bottom-up development approach ^294^.

The success of cybersecurity will equally depend on good governance that ensures compliance with safety regulation by all parties.

***Sustainability***. The need for financial sustainability to support capacity and infrastructure for data sharing is underscored ^167, 169^. Efficient pooling of resources for integrated data sharing platforms and joint funding application for data sharing initiatives by research partnerships have also been recommended ^295– 298^. Other proposed funding mechanisms include the establishment of foundations or charitable trusts to stimulate donor support towards public benefit, and a model involving a shared cost approach by partnering with governments, non-profit organisations and commercial entities ^299^.

Researchers have recommended that the sustainability of the databank must be determined from inception ^104, 117^. Ensuring sustainability will include consistent application of the policies throughout its lifespan including promoting scientific and ethical integrity ^47^. Discontinuation or change of ownership or eventual disposal of data should form part of the sustainability plan ^112, 117^. Obtaining appropriate liability insurance for a databank may be a way of ensuring its sustainability ^252^. There are potential opportunities for public-private-partnerships for public good, which may involve private sector use of public data for research or the integration of private sector data in public data, or public-private partnership for innovation and development ^300^. On the other hand the challenges to data sharing for commercial use mostly pertain to issues of social licence and public distrust and limited oversight of commercial data, data ownership, intellectual property, commercial secrecy, insufficient transparency, and profiteering ^300^.

Importantly, ensuring the sustainability of the databank must assume the qualities of a resilient system. Such a system is defined by its capacity to proactively adapt to changes and challenges to its daily operation and sustenance ^301^. This may also involve collaborative learning and stakeholder involvement as vital prerequisite pillars ^302^. Human capital and its adaptive capacity to such innovation will require digital literacy of platform users as well access to technology ^303^. These attributes help to create a system that is flexible, and adaptable to variabilities and improvisations ^304^. Moreover, a protocol to develop a resilient system that responds to cross country population health needs are described ^301^. Role clarification of the different stakeholder groups specified ^121^ is equally essential to the sustainability of databanks. Further requirement for system’s sustainability and adaptive capacity have been richly described and graded in terms of human capital and financing raking ^305– 308^.

***Data harmonisation***. There are exemplary data sharing repositories in Africa, but these platforms have different levels of information technology, different data structures and largely operate parallel to each other. Integrating such databases may require a harmonised data sharing platform.

Harmonisation is complex. Townsend ^309^ argues that it can be achieved through a bottom-up approach. This proposition is premised on consortia and stakeholders’ capacity to work together to find common grounds, policies, and solutions. An example is made about the success of GA4GH and P3G consortium, and the same can be said about H3Africa deliberative and accountability mechanisms ^42, 310, 311^. 

Other than government agencies, public and population health data in Africa predominantly sits with non-governmental organisations, charities, and research and academic institutions. Furthermore, the repositories may be institutional such as a university; governmental holding of administrative, service delivery or surveillance data; discipline specific repository ^193^. These institutions are predominantly donor funded and thus, expected to make data available to initiatives that serve public interest.

There are technical challenges to integrating and managing multi-disciplinary data from diverse jurisdictions. These include data dispersion, provenance and heterogeneity ^46^. This triple challenge arises from the thousands of possible data sources across the continent on different public and population health topics varying in scope and scale. These data are also collected using different methodologies, formats and data management protocols ^46^. The issue of dispersion may be addressed by harmonising and augmenting routine national survey and encouraging in-country groups and independent researchers to adopt existing tools where necessary and store data in a secured and legal repository. To reduce heterogeneity, similar methodologies may be promoted among contributors to repository with incentives to promote contribution. The submission of metadata describing data elements used for each project will promote accurate utility and integration. Dealing with these challenges can be done in a manner that does not create unintended ethical breaches such as uncontrolled or unauthorised re-identification or disclosure of participant information. Other challenges and opportunities of an integrated system are presented by Shah and Khan ^312^ and Jones *et al.* ^71^.

## Discussion and Conclusion

This article focused on global data sharing practices, and the development of databanks in Africa. The various documents reviewed, and interviews conducted with African stakeholders, offer insights on key challenges to data sharing and databanks. In addition, this research showcases existing opportunities that may be leveraged to develop a multi-consortia public and population health data sharing platforms in Africa, and similar contexts in LMICs. Specifically, African governments can learn from the mistakes of high-income countries on data sharing practices and tap into their positive and practical strategies that may enhance efficient development of integrated databanks in the region.

There are already, best practice platforms in Africa. Initiatives such as the INDEPTH, H3Africa Consortium and the African Academy of Science’s DELTAS programme are developing capacity in several research institutions across the continent. Some of these initiatives not only provide exemplary data sharing guidelines in Africa, but also aim to shift the role of African researchers from being mere data collectors or community brokers to becoming active leaders capable of enhancing scientific growth in Africa ^2, 5^. Yet, we noted various structural, individual, and contextual challenges that may hinder data sharing in Africa. In addition, it is evident that genomic data sharing dominates the scientific world globally and Africa in particular. There is need to address existing factors that hinder data sharing as discussed above and incorporate genomic data with other public health data to enhance scientific benefits in public and population health.

Establishing an integrated databank in the African region is increasingly becoming a matter of when and not if. Bold regional and global treaties may be needed to ensure safe and secure uptake of digitally available data. This includes the continuous development, monitoring and governance of ethical and operational standards in response to data access and proliferation requirements to protect the privacy, security, safety, and anonymity of data contributors.

The rapid growth in human subject or tissue databanks and sharing facilities gives urgency for national regulatory bodies to create guidelines and policies on data management and sharing ^110^. Inadequate, or the absence of, such policy guidelines is a major setback in most LMICs, and Africa. Development of databanks is also an evolving area with the rising scope, scale and complexity of emerging data and data sources ushering novel questions around ethical principles ^10, 155, 242, 313, 314^. Additionally, incoherence of national laws and regulations coupled with varying levels of adherence to laws does not always translate to moral use of data nor offer a guarantee for public trust ^315^, hence the need for continuous development and oversight.

The implementation of dynamic consent and opt-out options for routine health service users at the point-of-care may be a solution to accessing public data in a manner that respects the autonomy of the patients or research participants. In the absence of an integrated databank, opt-out option remains an important ethical consideration with the rise in clinical audit research studies to measure quality of care ^26, 316– 319^.

Our research’s heavy reliance on experience from sharing of genomic data and lack of sufficient African studies in the literature is notable. This was due to the availability of publications on genomic data sharing and limited studies focusing on data sharing experience in Africa. The study does not cover the use of data integration for precision medicine from the Global North, which has its own specific ethical complexities already presented by Browman *et al.* ^235^. Furthermore, the findings and recommendations reported in this article, however, do not create a one-size-fit-all solution for Africa. Instead, they provide considerations on how to harness Africa’s opportunities for safe and secure optimisation of its available data. Africa lags behind in all essential public engagements required to build integrated databanks, as we found no study exploring the view of African populations on data sharing and databank governance. We suggest the use of various targeted surveys on various groups or researchers working on specific health research such as malaria, HIV, or genomic studies as consultative tool to establish public opinion on data sharing. 

There is also a need to reconsider consenting tools and processes to include follow-up clauses and mechanisms including the use of appropriate technologies. To this end, others have suggested the addition of an exclusion clause in the information sheet and consent form ^29^. This proposition resonates with recommendations that privacy protection policies should serve all dynamic interests of its stakeholders ^53^. This article also recognises the multitude of concurrent policies and regulations governing issues of consent, intellectual property, and confidentiality.

The African Union should consider developing multilateral privacy and data governance policies and framework like existing European Union and OECD treaties on data sharing or other Safe Harbour arrangements described by Dove *et al.* ^245^. This may be useful to address jurisdictional barriers and efficient resolution and monitoring of matters of registration, compliance review, recognition, monitoring and enforcement, public participation, and general operations and guiding principles.

The growth in data science technical expertise on the continent ^320^, efficient infrastructure management ^321^ and proficiency in scaling-up innovations could be harnessed to develop integrated databanks ^320^. Policies for data sharing will not be realised without dedicated funding and monitoring mechanisms. Funder requirements for the sharing of data are unethical if this cannot be done safely and meaningless if the infrastructure and skills to manage shared platforms is not developed. At the research project level, funding to ensure good meta-data is provided to enable meaningful sharing is needed. Investment in the sharing super structure, both technical and human, is required. The opportunity of developing an integrated databank may be best managed through benefit from big ethics structure of safe harbours. We also recommend a hybrid harmonisation approach ^322^. Blockchain technologies can be used to control access to data. Key informant interviews with African scientist suggests that most would like to participate in future use of their data if given the opportunity.

Public concerns about data sharing are viewed as conditions for sharing. Fortunately, there is a growing array of mitigation measures to address these concerns in partnership with the community. This takes cognisance of differences in the level of these concerns by socio-demographic characteristics. Fortuitously, a lot of the concerns are mutable with greater transparency and communication. Others have noted that healthcare providers are more likely to help individuals appreciate and participate in data sharing initiatives ^323^. Further classification into broad groups is made based on their concern about data sharing and who to trust with shared data ^323^. 

Exploring facilitators and barriers in African populations is paramount to future success particularly in the context of who holds the data, and role of socio-economic, cultural, and religious values in data sharing participation. The information will help establish public communication and in developing a platform that is responsive to the will, aspirations, and concerns of African populations platform. Risks posed by data sharing to different groups need to be explored and measures to increase protection require more investigation ^234^.

Other general recommendations are listed below, while specific recommendations to specific challenges and risks are presented in Table 1.

1. Developing a utilitarian integrated multidisciplinary databank for African may be feasible by harnessing the increasing data science technical expertise and strategic collaborations in the continent, together with the proliferation of cloud technology and concomitant reduction in cloud computing infrastructural costs and maintenance burden ^320, 321^.
2. Overall, Africa is well placed to advance in data integration given the wealth of global lessons to leverage. While there is opportunity to build the databank through integration and harmonisation of existing national surveys, HDSS datasets, biobanks, routine health service and administrative data, disease specific registries and notification systems, there are also lessons from prospective digitally enabled African multi-country surveys to build on ^324^.
3. An integrated African public and population health databank may be built on familiar and aptly described health system governance principles ^325^. The principles include strategic vision, rule of law, transparency, participation and consensus orientation, ethics, accountability amongst others. These principles are in line with the values for data sharing classified into two groups: substantive (e.g. harm minimizations, social justice and public benefit), and procedural (e.g. transparency engagement and reflexivity) ^326^.
4. A hybrid developmental approach that combines the benefits of bottom-up and top-down approaches should be explored.
5. African multi-consortia engagements initiatives may be a starting point to harness big datasets, technical capacities, institutional knowledge, policies, operational guidelines, governance mechanisms, strategic partnerships, and social licences and capital.
6. Our findings support the growing call to rethink the process and requirements for informed consent ^26, 316– 319^. Such efforts should seek to develop mechanisms that may allow a gradual build-up of data with appropriate permission for an integrated database.
7. Considering the wealth of data that already exist and their potential to be integrated to address regional public health challenges, extensive stakeholder engagement may be needed to decide how to manage the consent to use legacy data for future research as well as new approaches to future data collection. Such engagement may include the establishment of an inclusive stakeholder committee to generate recommendations for open dialogues and refinement. Other approaches have been used ^49, 53^.
8. Interventions should be developed to address known concerns about data sharing especially among underrepresented populations.
9. Attention should be paid to the issue of data quality in Africa through capacity building initiatives. This calls for both encouragement and making the provision of quality data an obligatory requirement ^80^ with support mechanisms. Additional bioinformatics training or incorporation of relevant skills development into training curriculum is also recommended ^327^.

**Table 1.** Specific considerations and recommendations.

Themes and sub-themes	Considerations (challenges and risks)	Recommendations
A) DATABANKS		
	✓ Africa lacks integrated data banks the various data repositories e.g., HDSS sites, H3Africa and DHS are not harmonised ^18, 42, 51^ ✓ There is limited oversight and unclear policies by government institutions and research ethics committees on data sharing and governance of databanks ^219^. ✓ Ethical, legal and social implications of secondary data sharing are mostly unresolved ^110, 328, 329^. ✓ Public fear of loss of privacy or confidentiality breach, data misuse and abuse ^56, 58, 59^. ✓ Poor communication on data use leads to distrust from participants ^60, 61^ ✓ Insecurity, growing cyber-attacks, fear of using the internet ^87– 89, 149^, and dishonesty due to fear of stigmatisation ^83^. ✓ Researchers fear of possible loss of academic advantage and independence; loss of intellectual property ^70^.	➢ A need to develop integrated and harmonised databanks and frameworks for data sharing in Africa ^133, 140, 330^. Examples could be drawn from the Australian Population Health Network ^65^; the Canadian National Data Platform ^66^; and the UK’s Health Data UK ^67^. ➢ Develop policies on regulatory oversight and that enables collaborations ^47, 48^. ➢ A need to develop a harmonised agreement that respects the independence of separate entities while promoting robust and efficient cross-disciplinary research within the confines of national and international ethical and legal frameworks ^68^. ➢ Develop proper governance of databanks, quality management and sustainability ^47, 48^. ➢ Data custodians must adhere to ethical guidelines (e.g., privacy, trustworthiness) in data sharing ^83– 85^, and use or share the data for public good and social justice ^89, 149, 152, 331^. ➢ A need to improve on communication to research subjects regarding data sharing using strategies such as modular education approach ^90^; use of video ^91^, pictures and vignettes ^92, 93^. ➢ Need to conduct a public education on data reuse to promote trust and public participation ^78^. ➢ A need to collect rich metadata of each data set ^80, 81^. ➢ Other considerations are detailed in Wiehe *et al*., including identifying data sources/patterns, engagement with leaderships, ethical and regulatory compliance, etc. ^121^.
B) DATA PROTECTION LAWS AND GUIDELINES		
	✓ Limited to moderate data regulation and enforcement particularly in Africa ^92^. ✓ Other unaddressed issues include public view or perceptions of cross border data transfer ^120^.	➢ African countries without data protection policies must develop data protection policies by learning or borrowing from global models such as the UK Data Protection Act of 2018 ^94^, and examples from the African continent ^92^ ➢ Develop safe harbour privacy protection principles to address cross border regulatory bottlenecks, increase data sharing efficiency, and promote data harmonisation ^245^.
**Ethics** ** Committees** ** (EC)**	✓ lack of legal protection ^96^. ✓ Inability to reach quorum in decision making and inappropriate constitution of ethics committees (EC) ^152, 153^. ✓ Inefficiency or bias amongst its members ^99^. ✓ Lack of financial and administrative support to enable it to function smoothly ^332^. ✓ Social implications of data sharing often falls outside the ECs mandate ^50, 100, 101^. ✓ EC members’ poor familiarity with secondary data use, including laws governing cross-border transfers may be an impediment to safe data sharing.	➢ A need to build capacity of research EC to ensure consistent and efficient application of data sharing regulations ^333^. ➢ EC must be guided by global ethical guidelines including The Helsinki Declaration which provides guidance on data security, ethical principles and governance of data sharing ^102^. ➢ Other guidelines include: the Australian Guidelines on Human Biobanks and Genetic Research Databases ^103^; The OECD Principles and Guidelines for Access to Research Data from Public Funding ^104^; the Bermuda Principles ^105^; Fort Lauderdale Agreement ^334^ among others. ➢ The material transfer agreement (MTA) documents should include issues of data provenance, data quality assurance, meta-data and other requirements for accurate interpretation of data, intellectual property, informed consent, security and privacy terms etc. ^107, 335^. ➢ Develop Ethics waiver policies including setting up a central adjudicator of request when re- identification is necessary, and consenting is impractical. Examples include the Confidentiality Advisory Group (CAG) and the Public Benefit and Privacy Panel (PBPP) in England and Scotland respectively ^54^.
**Consenting**	✓ There are no clear guidelines for conducting informed consent ^336^. ✓ Complex use of data has make it difficult to differentiate between data collected for routine medical care and data collected for research ^49, 198– 201, 337^. ✓ Possible risk of patients and research participants being relegated to data donors, and negating the principles of autonomy and self-determination ^109^ ✓ There are unresolved issues on future use of data, including when participants want to opt in/out of studies ^111^. ✓ The scope of consenting is also not so clear in longitudinal studies, especially those involving minor ^62^, and parents may be reluctant to consent for minors.	➢ It is important to give people the opportunity to negotiate how others use their personal information ^338^. ➢ Researchers/Investigators must ensure that consenting process is a broad, continuous process and touches on data sharing clauses (data sharing now and, in the future), and ensure waivers permitting the use of de-identified data ^110^. ➢ Longitudinal studies should have follow-up mechanisms e.g. collecting additional identifiers for participants on the consent form to allow future re-contacting for further consenting ^156^. ➢ Research ethics committees should contend with emerging considerations of data stewardship such as the longer than usual data storage, sharing, re-identification and indeterminate future use of collected data ^26– 30^. ➢ Researchers must ensure that participants have enough information about their studies and consent options including a consent waiver, dynamic consent to opt in and/or opt out etc. ^111^. ➢ There is need to adapt existing software to facilitate data governance and participants’ control of their data ^52, 339, 340^. Examples include Fast Healthcare Interoperability Resources (FHIR), Sync for Science, Private Access, Patient Health Records (PHR) and Blue Button ^133, 239^.
**Data ownership**	✓ Laws and Policies on data ownership are not clear ^133^. For instance, patients have the right to request and retain their data. Similarly, clinicians have the right of data retention for clinical purposes, ✓ This lack of clarity on data ownership and custodianship is influenced by variations of what constitutes data – Data range from numbers to letters, symbols, idea, condition or situation ^341^.	➢ Data ownership should be governed by legal and moral obligations including trust and custodianship with variations in the right of access and utility by different stakeholders ^133– 135^. ➢ There is a need to adopt a non-exclusive ownership of data – whereby data ownership should be governed by legal and moral obligations. ➢ Data custodians must adhere to principles of respect for privacy and autonomy; reciprocity and feedback to stakeholders; acknowledgment and attribution to contributors; and, respect for intellectual property ^107, 311^.
**Intellectual** ** property rights**	✓ Data sharing may raise several issues to researchers, employers, and funders on: What are the legal rights in data? Who has these rights? And how does one with these rights use them to share data in a way that permits or encourages productive downstream uses? ✓ Some data repositories e.g., journals have strict measures that hinder access to data by those who cannot pay for it.	➢ There is need to develop a system and guidelines/templates for Intellectual Property that is guided by local intellectual property laws ^104, 114, 136^. ➢ Databank users are required to report back all publications and patents emanating from the data provided to them ^107, 117, 119^. ➢ Genomic databases are global public good and all humans should share in, and have access to, the benefits of databases ^342^. Similar views are shared in UNESCO’s International Declaration on Human Genetic Data ^343^. Thus, provide access to databases to anyone who rightfully demonstrates a need to access the data.
C) ENABLERS OF DATA SHARING		
**Stakeholder ** **and Community** **engagement**	✓ The concept of Stakeholder/community engagement is somewhat ambiguous, and there is lack of clarity of who must be included in the consultations ^344, 345^. ✓ Loss of trust may pose a risk to social licence ^315^. ✓ Unresolved situations like the continuous involvement of patients or study participants have the potential to weaken public trust and negates the principles of solidarity and social justice ^109^.	➢ Stakeholder consultation is an important strategy to promote equity, trust, transparency, autonomy and participation in data storage/sharing ^10, 109, 153^. ➢ Communication should be done with the required sensitivity to avoid ambiguity and misinterpretation ^153^ ➢ Community engagement should commence at the beginning of the project, to ensure feasibility and timely risks mitigation with stakeholders’ input ^109^. ➢ The consultation should clarify purpose of the data storage and sharing platform, roles and responsibilities, governance and accountability mechanisms, data protection, types of informed consent, benefit sharing, intellectual property, and data ownership. Exemplary framework can be drawn from H3Africa ^154^.
**Trust**	✓ Social licence may be misinterpreted as trust, which may be implied as informed consent to use information offered for research ^137^.	➢ In the case of big databanks, maintaining trust should be on-going and not a onetime checkbox activity. ➢ The engagements should also be cross cutting to involve other researchers, policy makers and funders, and not only research participants and communities ^112, 113, 140, 141^.
**Respect ** **for study** ** participants/** **groups**	✓ Issues may include where researchers do not disclose fully to participants on future use of data. ✓ Another issue would be not being clear during consenting time whether participants will be recontacted or not.	➢ Use of data should be in line with the scope of original informed consent provided by the research participants. ➢ The intention of the research is clearly stated during consenting/at the time of data collection including likely future use of the data ^112, 114^. ➢ In absence of specificities, broad consenting should be done to protect the research participants ^112– 114^. ➢ Elements of respect may include privacy protection and confidentiality; autonomy; data security; respect for individuals and group rights; ensuring dignity of participants; and, protection of life, wellbeing and welfare ^10, 102, 112, 119^. ➢ Re-contacting participants should of course, follow standard ethical principles including options on communication of findings or participant access to data ^117, 118^.
**Transparency**	✓ Providing patients or study participants with insufficient information on how data will be managed or shared ^83, 86, 152, 346^. ✓ unspecified secondary use of data ^104^. ✓ Giving multiple users access to data ^99, 104, 105^. ✓ Data misuse, identity theft and sharing data on the internet ^99, 101, 103, 119, 121^, and centralised database without sufficient safeguards ^99, 119^.	➢ Researchers must ensure participants are informed about how data will be shared and with whom ^53, 142^. ➢ Researchers must disclose to participants about monitoring policies and database governance, conditions framing access to data and data access agreements ^144– 146^. ➢ Also, disclose the role of patients and human rights advocacy groups involvement in providing oversight and supervision of the platform to ensure unbiased access and utilization of the databank ^148^. ➢ Ensure proper keeping and communicating sufficient records of operational activities including audits logs and trails ^86, 87, 149^.
**Incentivization** ** of data ** **contributors ** **and users**	✓ funding agreements, collaborative agreements, data sensitivity, privacy, giving up chance to publish, public critique, lack of data repositories and the absence of consent to share ^160, 165^. ✓ Fear of exploitation especially amongst researchers in low resources countries ^161^. ✓ Threat to intellectual property, professional value and economic benefits ^166^. ✓ The greater value placed on publications by institutions may also be discouraging data sharing ^164^.	➢ It is important for governments and funders to ensure capital and infrastructural development, and financing to promote research data sharing ^165, 167– 169^. ➢ Research institutions and researchers need to promote tangible reward in the form of reputational incentives and peer recognition including citation to enhance data sharing ^158, 170^. ➢ Make data sharing a requirement for project funding, journal publications, university tenue or promotion ^212, 270^. ➢ A need to develop clear data sharing policy that addresses the concerns of all stakeholders, including monitoring and reward mechanisms ^161, 173^. ➢ A need to promote diversity and inclusion of minorities and vulnerable groups ^56^ and international partners in data sharing ^178^. ➢ Develop open data badges – which is a tested intervention to improve data sharing ^171, 179^.
**Funders and** ** researchers’ ** **position**	✓ Most researchers or scientists in Africa are hesitant to share their data largely due to lack of awareness of the benefits of data sharing. ✓ Lack of funding and limited provisions for data sharing. ✓ Few members of African ethics review boards are familiar with the concept of data sharing amongst other ethical issues discussed such as broad consenting	➢ We recommend proactive advocacy to ensure that the concept of data sharing becomes a mainstream consideration in national discussions of research management and governance ^70^. ➢ There are policies that illustrate that all data is public good, and all funded research should be shared. This includes the Wellcome Trust ^180^ and the USAID’s Policy on Development Data ^181^. ➢ A need to train researchers on data management, and the recruitment of dedicated support staff to document data and manage repositories ^155, 221, 253^.
D) GOVERNANCE AND VALUE-BASED IMPLEMENTATION		
**Policies and** ** Values**	✓ Most guidelines and regulations within Africa do not provide clear guidance on governance and how data and samples ought to be shared ^182, 183^. ✓ Lack of clear policies on data sharing may both frustrate researchers who want to share data and provide loopholes for those who are unwilling to share. ✓ Diminished confidence on government custodial of the data ^142^.	➢ Governments and research institutions in Africa must develop clear guidelines on data sharing and repositories. ➢ Create clear laws to govern re-identification and stronger sanctions and corresponding enforcement protocol for misuse of data ^133, 189, 190^. ➢ Establish proper governance by providing a guideline on who, how, when and under what authority datasets can be linked or merged ^83^. ➢ Develop a central policy and inclusive governance structure that promotes collaboration and participants ^133, 148^.
**Data ** **anonymization ** **and re-** **identification**	✓ There is also an increase in clinical audit of patient records for quality improvement practice and research without individual patient consent ^50, 198– 201^. ✓ Yet, data anonymization may be challenging when researchers or clinicians want to link medical data to make clinical decisions in future, or recontacting patients to obtain additional information. ✓ Growth of the database means anonymity will not allow linking datasets or to re-identify individuals in the database if there is ethical reasonability and lawful approval to re-identify the participants ^113, 119^.	➢ Researchers and data custodians must be aware of possible identifiers, which can be direct or indirect ^191, 209^. ➢ Data controllers must uphold to the consent given by patients or study participants, use of appropriate technologies, mechanisms and permission to promote pragmatic dynamic consenting processes properly described by Kaye *et al*. ^216^. ➢ Researchers must ensure that details on data reuse and protective measures are clearly stated in the informed consent, and inform participants when absolute anonymity is increasingly impossible to guarantee albeit highly preventable ^107, 191, 192^. ➢ It is important to adequately educate researchers and data custodians to ensure data privacy protection compliance as well as signing renewable confidentiality pledges ^153^. ➢ Data should be de-identified before it is shared ^310^
**Data Access**	✓ Most data sharing agreements are silent on the consequences of violating data access agreement ^234^ and rely on national regulations. ✓ Limited awareness and access to databanks available for secondary users ^219^.	➢ Develop clear data access agreements or guidelines on what the application can and cannot do with the data provided ^260^ as well as consequences of nonadherence to data access agreement ^234^. ➢ Data access should not negate the principles of autonomy, privacy, public interest and benefit, acknowledgment of data contributors, transparency, accountability and trustworthiness ^193^. ➢ Promote data access discussions during stakeholder and collaborative partnerships, including resource provisions to addressing the impediments to data sharing ^220^.
**Data access** **committees ** **(DACs)**	✓ Financial constraints and lack of sufficient oversight mechanisms ^240^. ✓ There is lack of clear definition of the relationship between DACs and biomedical research ethics committees (ECs) when conducting evaluations. ✓ Insufficient oversight mechanisms ^59^ ✓ Inequalities in terms of the composition of DACs- which may exclude important stakeholders ^242^. ✓ Conflict of interests between DAC members and other stakeholders ^242^.	➢ DACs must be provided with adequate funding to perform their roles ^240^ ➢ Develop clear guidelines and framework to guide functioning of DACs. ➢ A need to adapt to technological, scientific, data security, new data sources and research methodological advances and changes in public sentiments ^347, 348^. ➢ The need to have an oversight over DAC is recommended ^59, 240^. ➢ To address inequalities and curtail vested interests, DACs should be inclusive, global and transparent ^242^. ➢ DACs should be an independent committee without conflicts of interest or measure to evaluate and mitigate its internal risks ^240^.
E) DATA INFRASTRUCTURE, QUALITY, STORAGE AND SECURITY		
**Infrastructure**	✓ Many African institutions have limited infrastructure (spaces, inadequate equipment/ tools, power supply shortages, poor information technology) for data repositories and data sharing ^1– 3^ .	➢ There is a need to develop ICT infrastructure and efficient workflow; harmonised policies, guideline and operating procedure; data access policies and mechanism; and, government regulation and oversight ^349^. ➢ Other considerations include human and social capital, financial resources and governance ^350^. ➢ Developing an adaptive information technology enabled system. ➢ Ensure adequate financial resources to address the mentioned challenges.
**Data Quality**	✓ Some of the reasons why scientist do not reuse data include concerns about data quality; lack of awareness of benefits of big data; and, lack of technical capacity to use big data ^351^. ✓ Scepticism and self-doubt of quality of research may inhibit some researchers from sharing their data ^178^. ✓ Poor data quality in Africa is due to lack of infrastructure, inadequate skills and capacity amongst researchers as well as lack of guidelines on how data must be prepared or processed.	➢ Data custodians and Databanks must establish high quality threshold indicators for routine review and updating ^104, 112, 117, 248^. ➢ Data quality assurance should be documented, unbiased, open to review, factual and proportionate ^10, 104, 117, 119^. ➢ Researchers and data custodians must establish the contextual meaning of data to minimise misinterpretations. Example can be drawn from the H3Africa model ^42^. ➢ It is important to also offer data seal of approval to guarantee researchers that data will be stored in good quality, and consistent reuse while ensuring the trustworthiness of digital archives ^250, 251^. ➢ Regulatory licencing and oversight of databanks could also help ensure quality and accountability ^252^.
**Data storage &** ** Retrieval**	✓ Identification of anonymised data, increased risk of disclosing other data, misinterpretation of data for various reasons, malicious use of data, harm to the public posed by illegal disclosure and commercialization ^128, 253^.	➢ Cataloguing data in a consistent manner will promote harmonization and interoperability ^254^. ➢ African data scientists or custodians must draw from internationally accepted norms and standards to ensure compatibility ^104^. ➢ Data custodians (e.g. on online platforms) must ensure: metadata availability, discoverability, data standardization, quality assurance, storage, backup, migration, succession plan, legal status, access and terms of use and more shown in the table ^161, 255^. ➢ Develop an integrated system such as the Open Archival Information System (OAIS) for data management and sharing ^256, 257^. ➢ Databanks must store anonymised or de-identified data with additional safety and access control measures ^24, 113, 259, 260^; use individual unique identifiers ^153^ or aggregate datasets ^218^.
**Security**	✓ For cloud data- issue of integrity and exploitation of data by service provider and its employees ^222, 274– 276^, cloud attacks ^277^, ✓ User identity spoofing ^278^, ✓ Data tampering ^279^. ✓ Denial of service ^280^. ✓ Unlawful access to database and infiltration of the system ^278^. ✓ Danger of re-identification of de-identified data ^281^.	➢ The success of data security (including cybersecurity) will depend on good governance that ensure compliance with safety regulation by all parties. ➢ A need to develop policies on data security that mandate the custodians of data to protect it from abuse, unauthorised access and tampering, loss or unlawful disclosure ^272^. ➢ Privacy protection provide a notification in the event of breach of privacy due to unauthorised access, loss or disclosure of information in the care of a legal data custodian ^273^. ➢ Establishment of remote access controlled data centres, and good monitoring systems ^107, 283^.
**Sustainability ** **of databanks**	✓ Challenges to sustainability include the cost of maintaining a central databank, issues of social licence and public distrust and limited oversight of commercial data, data ownership, intellectual property, commercial secrecy, insufficient transparency, and profiteering ^300^. ✓ Funding constraints also have implications on data cleaning, analysis, storage, which may ultimately affect the data quality.	➢ Researchers must plan for sustainability of databank before their studies commence ^104, 117^. ➢ A need for consistent application of data policies throughout its lifespan including promoting scientific and ethical integrity on data ^47^. ➢ Governments and funders must increase financial sustainability to support capacity and infrastructure for databanks and data sharing ^167, 169^. ➢ There is also a need to invest in human capital ^305, 306, 308^. ➢ Other ways of ensuring sustainability of databanks is through obtaining appropriate liability insurance ^252^. ➢ Public-private-partnership in data management can improve for innovation and development and sustainability of databanks ^300^. A good example can be drawn from , the European Union’s Data Protection Regulation ^300^.
F) DATA HARMONIZATION		
	✓ Data repositories in Africa are disintegrated. Consortia are often not homogenously impractical to developing consortium specific data sharing guidelines. ✓ Many consortia have specific guidelines which may make it difficult to integrate data. ✓ Data repositories in Africa largely sits in research institutions or NGOs or generalist data repository that are not specific to any discipline; and project or programme specific repository ^193^.	➢ Develop an integrated multidisciplinary guideline that is flexible for public and population health. And which will allow multilayer data sharing for public good ^10, 133^ ➢ Develop stakeholder-centric ecosystems in terms of its principles and policies seeking to efficiently meet the needs of its members ^133^. ➢ Stakeholders must work together, through a bottom up approach, to find common grounds, policies, and solutions to harmonization challenges ^235, 309^. Examples include success of GA4GH, P3G and H3Africa ^42, 310, 311^. ➢ Develop a flexible guideline/policy interoperability and convergence between partners to facilitate collaboration and platform efficiency ^330^.

## Data availability

### Underlying data

Zenodo: Public and Population Health data sharing in Africa – views of academics and researchers, https://doi.org/10.5281/zenodo.5155880 ^352^.

This project contains the following underlying data:

De-identified transcripts of the interviews with the 24 key informants

### Extended data

Zenodo: Interview Guide Used in the Key Informant Interviews: Public and Population Heath data sharing in Africa - Views of Academics and Researchers https://doi.org/10.5281/zenodo.5168457 ^39^.

This project contains the following extended data:

Interview guide use in the key informant interviews

Data are available under the terms of the Creative Commons Attribution 4.0 International license (CC-BY 4.0).

## Footnotes

^1^“In general, anonymisation refers to the process of removing identifying information such that the remaining data cannot be used to identify any particular individual…Data would not be considered anonymised if there is a serious possibility that an individual could be re-identified, taking into consideration both: (a) the data itself, or the data combined with other information to which the organisation has or is likely to have access and (b) the measures and safeguards (or lack thereof) implemented by the organisation to mitigate the risk of identification.” ^195^. Anonymisation is also used in to refer to de-identified data that cannot be reversed ^189, 197^. HIPAA defines ammonised data as ‘health information that does not identify an individual….there is no reasonable basis to believe that the information can be used to identify an individual….” ^189^.

^2^“The degree to which an individual can be identified from one or more datasets containing direct and indirect identifiers” ^195^

^3^“The degree to which an individual can be identified from anonymised dataset(s)” ^195^
