# Supplementary material for: Considerations for an integrated population health databank in Africa: lessons from global best practices
Source: Wellcome Open Res. 2021 Aug 23;6:214. [Version 1] doi: 10.12688/wellcomeopenres.17000.1 (PMC8844538; doi:10.12688/wellcomeopenres.17000.1)
Supplement: Peer review history

# Peer review: Reviewer response for version 1

**DOI:** 10.21956/wellcomeopenres.18772.r47220

**Published:** 2022-02-14

**Authors:** Ushotanefe Useh

**Affiliations:** 1 Lifestyle Diseases Research Entity, Faculty of Health Sciences, North-West University, Potchefstroom, South Africa

This review sought to provide practical recommendations and requirements to support the development of a multi-consortia public and population health data sharing framework for Africa. This research seeks to inform a platform that will harnesses available resources, provide incentive, data sharing, and optimize the progress made by different research groups in Africa.

 This work was a narrative of over 655 documents, publications and policies on data sharing in public and population health. The authors also reviewed data protection laws and regulations as they affect different countries in the continent of Africa. They also provided recommendation in Table 1. Among those cited were those from South Africa, Kenya, Nigeria, Togo, Tanzania, Uganda and Zimbabwe.

 The study design is appropriate with both narrative review and interview of 35 key informants from African-led research capacity building programmes. Detailed procedure is provided in the study. A detailed Table and with specific consideration is provided.

 More than 9 recommendations were provided by the authors. These ranged from developing an integrated data bank, infrastructure development to capacity building initiatives.

Is the work clearly and accurately presented and does it cite the current literature?

Yes

If applicable, is the statistical analysis and its interpretation appropriate?

Not applicable

Are all the source data underlying the results available to ensure full reproducibility?

Yes

Is the study design appropriate and is the work technically sound?

Yes

Are the conclusions drawn adequately supported by the results?

Yes

Are sufficient details of methods and analysis provided to allow replication by others?

Yes

Reviewer Expertise:

Sociology of Health and Illness and Lifestyle Diseases

I confirm that I have read this submission and believe that I have an appropriate level of expertise to confirm that it is of an acceptable scientific standard.

# Peer review: Reviewer response for version 1

**DOI:** 10.21956/wellcomeopenres.18772.r45702

**Published:** 2021-09-07

**Authors:** Ejemai Eboreime

**Affiliations:** 1 Department of Psychiatry, Faculty of Medicine and Dentistry, University of Alberta, Edmonton, AB, Canada

This is very interesting and important article that attempts to identify the requirements and provide practical recommendations for developing a multi-consortia public and population health data-sharing framework for Africa. I believe this situates and compliments the ongoing debates about registration and repositories for global health research.

 I have a few minor comments for the authors' consideration.

**Methods:**

 It is clear that the authors conducted a multi-methods study (a narrative review and key informant interviews). However, this is not stated clearly in either method sections of the abstract and the main text. My initial thought as I read the article was that this was a narrative review, but as I progressed with reading, I found that interviews were also conducted. I believe stating clearly that two approaches were used, before describing both approaches separately would give readers an early understanding of the research approach used. Further to this, a line or two on how triangulation was conducted would be valuable.

**Discussion:**

 I think a line or two that incorporates this discussion into the interesting (similar) debates around global health research registration and repositories would be an awesome idea. There have very recently been questions as to the necessity or absurdity of this approach in global health initiated by an editorial of mine in the BMJ Global Health ^1^. This editorial sparked a commentary that highlighted challenges with centralized repositories for community-oriented research which make it an absurdity ^2^. It would be interesting for the author to throw some thoughts on how their findings are situated in these debates.

Is the work clearly and accurately presented and does it cite the current literature?

Yes

If applicable, is the statistical analysis and its interpretation appropriate?

Not applicable

Are all the source data underlying the results available to ensure full reproducibility?

Yes

Is the study design appropriate and is the work technically sound?

Yes

Are the conclusions drawn adequately supported by the results?

Yes

Are sufficient details of methods and analysis provided to allow replication by others?

Yes

Reviewer Expertise:

Health systems, Global health research, Implementation science

I confirm that I have read this submission and believe that I have an appropriate level of expertise to confirm that it is of an acceptable scientific standard.

## References

[1] : A priori registration of global health research-necessity or absurdity?. *BMJ Glob Health* .6(5) : 10.1136/bmjgh-2021-006199 10.1136/bmjgh-2021-006199 34001522PMC8130736

[2] : The absurdity of research registration for community-oriented knowledge coproduction. *BMJ Glob Health* .2021;6(8) : 10.1136/bmjgh-2021-007040 10.1136/bmjgh-2021-007040 34413079PMC8378344
